# Dynamics of SARS-CoV-2 infection over two epidemic waves in Manaus, Brazil: A serological study of seven thousand blood donors

**DOI:** 10.1371/journal.pone.0308319

**Published:** 2025-01-15

**Authors:** Neal Alexander, Christopher Dye, Michael P. Busch, Lewis Buss, Carlos A. Prete, Oliver J. Brady, Paul Mee, Claudia M. M. Abrahim, Myuki A. E. Crispim, Allyson G. da Costa, Tassila Salomon, Philippe Mayaud, Márcio K. Oikawa, Nuno R. Faria, Ester C. Sabino

**Affiliations:** 1 Department of Infectious Disease Epidemiology, MRC International Statistics and Epidemiology Group, London School of Hygiene and Tropical Medicine, London, United Kingdom; 2 Department of Zoology, University of Oxford, Oxford, United Kingdom; 3 Vitalant Research Institute, San Francisco, CA, United States of America; 4 Department of Laboratory Medicine, University of California, San Francisco, CA, United States of America; 5 Faculdade de Medicina da Universidade de São Paulo, Instituto de Medicina Tropical, São Paulo, Brazil; 6 Department of Electronic Systems Engineering, Universidade de São Paulo, São Paulo, Brazil; 7 Department of Infectious Disease Epidemiology, London School of Hygiene and Tropical Medicine, London, United Kingdom; 8 Fundação Hospitalar de Hematologia e Hemoterapia do Amazonas (HemoAM), Manaus, Brazil; 9 Faculdade de Ciências Médicas de Minas Gerais, Belo Horizonte, Brazil; 10 Faculty of Infectious and Tropical Diseases, Department of Clinical Research, London School of Hygiene and Tropical Medicine, London, United Kingdom; 11 Center of Mathematics, Computing and Cognition, Universidade Federal do ABC, Santo André, Brazil; 12 MRC Centre for Global Infectious Disease Analysis, J-IDEA, Imperial College London, London, United Kingdom; University of Ilorin, NIGERIA

## Abstract

**Background:**

Most longitudinal studies of COVID-19 incidence have used unlinked samples. The city of Manaus, Brazil, has a blood donation program which allows sample linkage, and was struck by two large COVID-19 epidemic waves between mid-2020 and early 2021.

**Methods:**

We estimated the changing force of infection, i.e. incidence in susceptible individuals. Seroconversion was inferred by a mixture model for serial values from the Abbott Architect SARS-CoV-2 nucleocapsid (N) IgG assay. We estimated the number of suspected COVID-19 hospitalizations arising from each infection over calendar time.

**Results:**

Whole blood donations between April 2020 and March 2021 were included from 6734 people, 2747 with two or more donations. The inferred criterion for seroconversion, and thus an incident infection, was a 6.07 fold increase in N IgG reactivity. The overall force of infection was 1.19 per person year (95% confidence interval 1.14–1.24) during the two main waves. The estimated number of suspected hospitalizations per infection, was approximately 4.1 times higher in the second wave than in the first.

**Conclusions:**

Serial values from this assay can be used to infer seroconversion over time, and in Manaus show a higher number of suspected COVID-19 hospitalizations per infection in the second wave relative to the first.

## Introduction

COVID-19 is a respiratory and multi-organ illness caused by infection with the severe acute respiratory syndrome coronavirus type-2 variant (SARS-CoV-2) which emerged in late 2019 and was declared a pandemic in March 2020 [1]. As of 8 April 2022 over 494 million COVID-19 cases and over 6 million deaths have been reported worldwide [2]. Brazil is one of the worst affected countries with over 30 million cases and 0.6 million deaths reported to the surveillance system [2]. The city of Manaus in the Brazilian Amazon State suffered two distinct and damaging waves: the first peaking in May 2020, and the second around New Year 2021, coinciding with the emergence of the P.1 (gamma) variant of concern [3].

Donated blood samples are used for surveillance of HIV and other infections including SARS-CoV-2 [4–6]. If serial samples from the same donor can be linked then an incident infection can be inferred from seroconversion, enabling the estimation of the force of infection, i.e. “the rate at which a susceptible person acquires an infection” [7]. The objectives of this study were to use sequential values from the same antibody assay in repeat blood donors to estimate seroconversion and hence the force of infection of SARS-CoV-2 in the city of Manaus and then to compare the ratio of incidence of suspected COVID-19 hospitalizations to the estimated force of infection between two peaks of incidence in the city.

## Methods

### Study site and population

Study participants were repeat blood donors presenting at the Fundação Hospitalar de Hematologia e Hemoterapia do Amazonas (HEMOAM) in Manaus. This center stores residual blood donation samples for six months, and we retrieved stored samples from April to June 2020, with samples selected prospectively in subsequent months. Samples were selected consecutively beginning from the second week of each month. There were no further inclusion criteria. Exclusion criteria were missing data for age or sex. We did not perform a formal sample size calculation. Repeat samples from the same donors were linked via a donor code number. The current study did not use identifying information such as names or addresses. The first donation was 1 April 2020 and the last was 16 March 2021. The data were first accessed for the purpose of the current study on 3 February 2021. Overall, 9,987 samples from 6,734 blood donors were included in this study.

### SARS-CoV-2 IgG assay

The study used aliquots of plasma samples stored at -20°C after testing for other infections at HEMOAM. Samples were tested on the Abbott Architect instrument (Abbott Park, IL) using a chemiluminescent microparticle immunoassay (CMIA) for detection of IgG antibodies against the SARS-CoV-2 nucleocapsid protein (N) [8]. Assays based on S (spike) and RBD (receptor-binding domain) [9] were not yet available in our context. Chemiluminescence is measured as a relative light unit (RLU), and the presence of IgG was determined by “comparing the chemiluminescent RLU in the reaction to the calibrator RLU, which is calculated by the system as an Index (S/C)” [10], this being a signal-to-calibrator ratio [11].

### Statistical methods

Although the IgG assay is intended for qualitative use [10], we analysed the S/C values quantitatively. According to the manufacturer, a test result is positive if the S/C exceeds a predefined threshold. However, to be able to detect reactivity boosting in seropositive individuals consequent to primary infections as well as reinfections [12], we worked with the fold-change in S/C between serial donations instead of the absolute S/C value. A two-component normal mixture model was fitted to the distribution of the logarithm of fold changes in S/C, using the EM (expectation-maximization) algorithm [13]. Exploratory analysis showed a distribution which was clearly bimodal (Figure A in S1 File), so a null model with a single component was not considered. To discriminate between the two components of the distribution, the threshold fold change was taken to be the value where the probability densities are equal. Values above this threshold are taken to indicate seroconversion. The rate of such inferred seroconversions was then estimated as a function of the initial S/C value using a binomial regression model. Bearing in mind that the times of the event (inferred seroconversion) were not observed exactly, a model with complementary log-log link was used, with the logarithm of the time between the two consecutive donations as an offset [14]. This model then estimated rate ratios for age group and sex. The seroconversion rate was then fitted as a smooth function of the initial S/C value, specifically a B-spline [15] with five degrees of freedom. This was used to help set a criterion for seroconversion in terms of the absolute S/C value. The final definition of seroconversion was either the specified fold change, or a change from below to above the absolute value.

Using this combined definition, we estimated associations between age and sex and the force of infection, i.e. the rate at which susceptible people acquire infection [7]. This was done using the same type of binomial regression model as before, but with the post-seroconversion periods removed from the denominator on the grounds of not being at risk. Given that SARS-CoV-2 was not present in Manaus before 2020 [16], an initial negative donation was added to the dataset for each person. The date for this imputed donation was 1 March 2020. The S/C value of this imputed donation was arbitrary because it was not used to calculate fold changes. To allow for possible multiple pairs of donations within the same person, the standard errors were inflated using the “sandwich” estimator [17]. In order to estimate changes in the force of infection over calendar time, a Monte Carlo approach was used. For each simulation, the unobserved actual time of seroconversion for each donor was assigned a value uniformly random between the two ends of the interval defined by consecutive donation dates [18]. This was done 1,000 times, with a B-spline with five degrees of freedom being fitted to the weekly rates. We used Poisson regression because, within the assumptions of this analysis, the event times have been specified. The mean and confidence interval for the weekly rates were then calculated as summary statistics across the 1,000 simulations. The implied seroprevalence at week *j* was estimated from the weekly rates *λ*_*i*_ as $1‐\prod_{1}^{j}$ *e*^-*λi*^., i.e. one minus the probability of remaining infection-free over all the weeks in question.

The force of infection was compared to the incidence of suspected COVID-19 hospitalizations in the Brazilian Ministry of Health’s database on severe acute respiratory syndrome (in Portuguese, *síndrome respiratorio agudo grave* or SRAG), also called SIVEP-Gripe [19,20]. The clinical case definition requires all four of the following criteria to be met: (i) fever, including self-reported; (ii) cough or sore throat; (iii) dyspnea or O_2_ saturation <95% or respiratory discomfort; and (iv) hospitalization or fatal outcome [21,22]. Although this definition includes deaths of people that were not necessarily hospitalized, for simplicity we refer to such cases as suspected COVID-19 hospitalizations. They were included from the municipality of Manaus, with final classification as COVID-19 or unspecified (codes 5 and 4), as hospitalizations with unspecified etiology during the pandemic are likely underreported COVID-19 cases [23]. For each day, the rolling daily average of hospitalizations across the previous week was calculated. This rolling average was divided by the estimated daily incidence, calculated as the product of the daily force of infection and the proportion estimated susceptible (1 minus the seroprevalence). For graphical comparison, the rolling average of suspected COVID-19 hospitalizations was lagged by one week to represent the incubation period [24]. Finally, a thin plate spline was fitted through this ratio to facilitate comparisons between waves.

### Ethics

This project was approved by the Brazilian national research ethics committee, CONEP CAAE—30178220.3.0000.0068, who waived the need for informed consent for testing and analyses of coded residual blood donation-derived samples.

## Results

**Fig 1:**
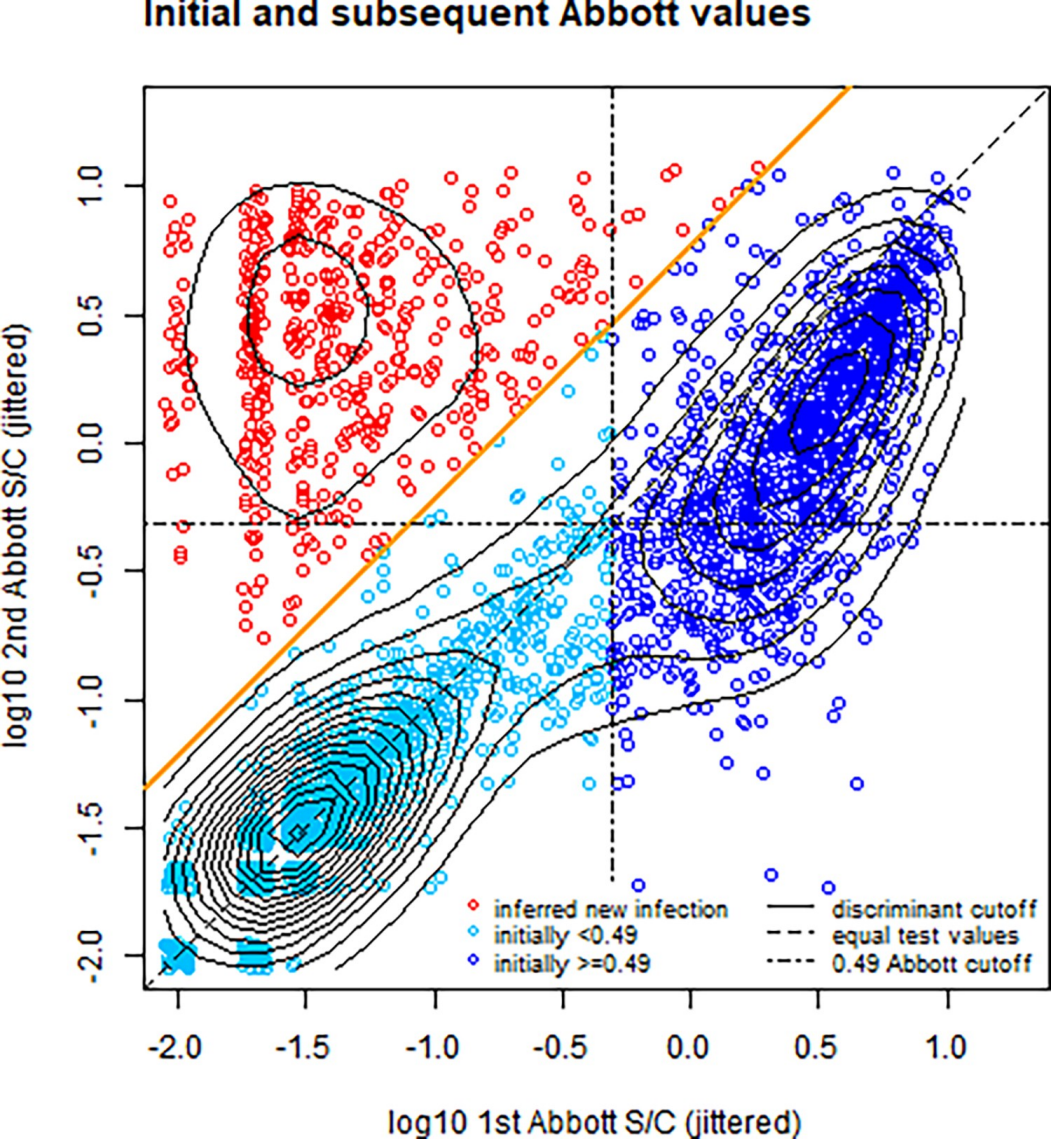
Sequential Abbott S/C values. Each point shows paired values from the same donor, and the contours are of probability density. The red points, above the solid orange diagonal line, have fold changes greater than the threshold value in Figure A in S1 File, i.e. are inferred seroconversions. Those below the orange diagonal line have a bimodal distribution. The vertical and horizonal dashed lines show S/C = 0.49. So, for example, those to the left of the vertical dashed line are defined as initially negative. Dark blue points, with first S/C ≥0.49, are defined as positive on the first donation, with any increase to the second donation being less than the threshold for fold change. Light blue points are negative for both timepoints, except for a small proportion in the right triangle below the orange line but in the upper left quadrant: These are above the fixed positivity threshold but with a low fold change. The contours show that the blue points are segregated into a bimodal distribution, shown as light and dark blue.

**Fig 2:**
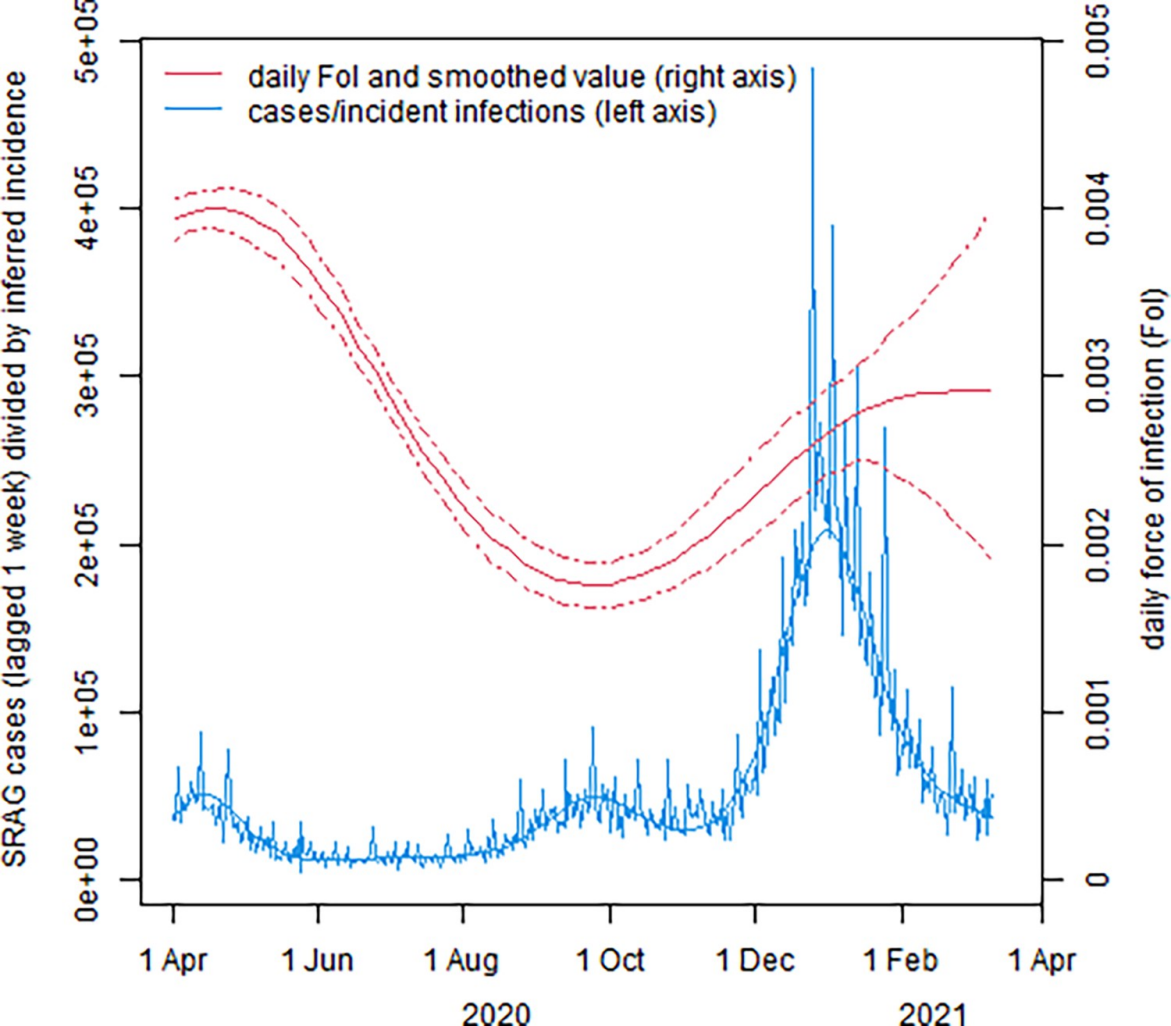
Force of infection over calendar time. The right axis, and the red line (red), show the daily force of infection estimated from within-person changes in Abbott S/C values. Dashed lines are 95% confidence limits. The blue line is the rolling daily average, over one week, of suspected COVID-19 hospitalizations in the SIVEP-Gripe database, lagged by one week [24] to reflect the incubation period, with a smoothed line in the same color. The ratio of the peak of the smooth line in the second wave to the peak in the first wave is 4.1.

Participants’ characteristics are shown in Table 1. The bimodal distribution of changes in Abbott S/C between consecutive samples is shown in Figure A in S1 File. The upper part of the distribution are changes from which we infer infection, including first infections and any with reinfection-induced boosting. The fitted threshold fold change for inferring seroconversion was 6.07. The initial and subsequent Abbott values are shown in Fig 1. Figure B in S1 File shows that the rate at which donors experienced this magnitude fold change in S/C dropped sharply as a function of the initial value. For an absolute cutoff value, this supports a value close to the lower end of the manufacturer’s “grey area” for positivity, which is 0.49 [8]. Table 2 shows the ratios of force of infection under the combined definition of seroconversion, i.e. either above the threshold fold change (which captures reinfections), or crossing the absolute value (0.49) (which identifies primary infections). The rate ratios for age are generally close to the null value (1), and all the confidence intervals cross this value. The changes over time in force of infection are shown, in relation to incidence of severe acute respiratory syndrome (SRAG), in Fig 2. Aggregating the force of infection over time gives the seroprevalence shown in Figure C in S1 File. The estimated number of suspected COVID-19 hospitalizations per infection was approximately 4.1 times higher in the second main peak of force of infection (i.e. highest part of the second wave), from December 2020 to January 2021, than in the first, in April 2020 (Fig 2). This suggests some combination of i) higher transmissibility and ii) a higher proportion of cases being sufficiently symptomatic to reach the database. Another explanation could be that the observed pattern is an artefact of a systematic difference in reporting of clinical cases between the two waves, i.e. cases of equal severity having different probabilities of being present in the database. However, the ratio of deaths to suspected COVID-19 hospitalizations was similar between the two waves (Figure E in S1 File), which tends to suggest that such an effect was not substantial.

**Table 1:** Participants’ characteristics.

	With a single donation	With two or more donations	Overall
Number of people	3987	2747	6734
Age at first donation in the current study: median years (interquartile range)	33 (24–42)	34 (25–43)	34 (25–42)
Sex: number (percent)			
Female	1024 (25.7)	544 (19.8)	1568 (23.3)
Male	2766 (69.4)	2105 (78.6)	4871 (72.3)
Number with missing age and sex (percent)	197 (4.9)	98 (3.6)	295 (4.4)
Date of first donation: median (interquartile range)	10 Sep 2020 (6 Jul 2020–15 Dec 2020)	16 May 2020 (24 Apr 2020–15 Jun 2020)	7 Jul 2020 (14 May 2020- 15 Oct 2020)
Numbers of donations per person (percent)			
1	3987 (100)	-	3987 (59.2)
2	-	2303 (83.8)	2303 (34.2)
3	-	385 (14.0)	385 (5.7)
4	-	56 (2.0)	56 (0.8)
5	-	3 (0.1)	3 (0.04)

**Table 2:** Force of infection by age and sex. The event is inferred seroconversion based either on fold change, or change from below to above the absolute threshold. The person-years includes time between donations, and also from an inferred negative value to the first donation. In order to estimate the force of infection, periods after the first positive result for each person are not included, on the basis of not being at risk. People with missing age or sex are not included. Note that the overall rate cannot be obtained by simple division of the events and person-years between donations, because the exact times of the events were not observed.

	Inferred seroconversion events	Person-years between donations	Rate ratio (95% CI)
Age in years at latest donation			
16–19	248	255.0	1
20–29	969	1069.7	0.92 (0.78–1.10)
30–39	875	951.4	0.94 (0.79–1.12)
40–49	685	729.8	0.98 (0.82–1.16)
50–59	272	297.9	0.93 (0.76–1.14)
60–69	52	66.2	0.81 (0.59–1.13)
Sex:			
Female	646	852.5	1
Male	2455	2517.5	1.35 (1.23–1.49)
			Rate per person-year
Overall	3101^a^	3370.0^b^	1.19 (1.14–1.24)

^a^Of which 3073 inferred by crossing the fixed threshold, 434 by fold change, and 406 by both.

^b^Of 3926.4 person-years, 388.7 (9.9%) were excluded for not being at risk due to previous seroconversion, and another 167.7 excluded due to missing age or sex data, yielding this total of 3370.0.

## Discussion

Seroconversion can be used to estimate incidence of infection [25,26] and as an endpoint in vaccine trials [27,28]. In this study, we used serial blood donations in order to estimate the force of infection over time in Manaus, the largest city in Brazil’s North Region, which suffered extremely high burden of COVID-19 in 2020-21 [29,30]. The ratio of new hospitalizations to the estimated force of infection in the second wave peaked at approximately 4.1 times the value in the first wave [31]. The higher ratio in the second peak could be due to higher transmissibility the Gamma/P.1 variant which dominated this wave [31]. Another possible explanation is that people at higher risk did not sustain initial risk mitigation behaviors, leading to a higher proportion of cases reaching the SIVEP-Gripe database.

Although specified for use as a qualitative assay [10], previous studies have established that numerical S/C values of the Abbott Architect assay were useful in clinical management [32]. They have also been used for monitoring vaccine response, based on antibody to the spike protein [33], rather than to the N) protein as in the current study. We found a clear bimodal distribution in the fold change of the S/C value of the Abbott Architect N IgG assay between successive blood donations, and infer an incident infection as either a fold changes in S/C in the upper peak of this distribution (which allowed for ascertainment of reinfections), or a change from negative to positive in terms of absolute value. For the latter, we chose a cutoff in accordance with the latitude specified by the manufacturer [34]. We found it most informative to choose a cutoff lower than the default, as other studies have also concluded [35,36].

Other studies with repeated surveys using either the Abbott Architect assay [37–40] or other assays [41], have been carried out among blood donors. However, these studies were cross-sectional at multiple time points, with any serial samples from the same donors being unlinked, so were not longitudinal in the strict epidemiological sense [42], thus did not directly estimate the force of infection. Beyond blood donors, cohort studies based on seroconversion have been carried out among health workers in Chicago [43] or a mixture of general public and other individuals in Northern Ireland [44]. The latter did not estimate incidence, but the former estimated a force of infection (incidence in those initially seronegative) of 4.25 per 10,000 person days, or 0.155 per year, less than the estimate of 1.19 per person year in the current study.

One advantage of using blood donors to estimate incidence of infection is that they do not need to be specifically recalled for serial measurements. On the other hand, a limitation is that the donors in this study were not representative of the population in many respects and a previous study has shown that repeat donors differ from first time donors [45]. In particular, men comprised a large majority (76%) of the current cohort, and experienced a higher force of infection. Cohort effects may have been present, such as exposure risk varying with the calendar date of first donation. Limited information is available on this, but the values of the Abbott test did not vary markedly between early and late entrants (Figure D in S1 File). Another limitation is that no adjustment has been made for waning of the antibody response [29,46,47], so infections are likely to have been missed due to the time elapsed between serial donations, which would underestimate final attack rate as calculated previously using serial cross-section surveys [48,49].

In conclusion, the current study demonstrated that fold changes in S/C values from the Abbott Architect N IgG assay to measure SARS-CoV-2 antibodies have a bimodal distribution, with the threshold between the two components of the distribution being a possible criterion for seroconversion resulting from first infections and reinfections. Applying this to blood donors in the city of Manaus in 2020–21 showed two peaks in force of infection, with the latter associated with a greater number of suspected COVID-19 hospitalizations per inferred incident infection. The results are likely to be generalizable to other locations which experienced two waves of similar variants. The approach may be applicable to other assays which provide a dichotomized outcome based on a continuous underlying index.

## Supporting information

S1 Checklist (DOCX)

S1 File Additional figures. (DOCX)

S1 Table Checklist of items for reports of cohort studies, according to the STROBE statement. (DOCX)
