# Supplementary material for: Dynamics of SARS-CoV-2 infection over two epidemic waves in Manaus, Brazil: A serological study of seven thousand blood donors
Source: PLoS One. 2025 Jan 15;20(1):e0308319. doi: 10.1371/journal.pone.0308319 (PMC11734911; doi:10.1371/journal.pone.0308319)

Incidence of SARS-CoV-2 over time in a cohort of serial blood donors in Manaus, Brazil

**Supporting Information: S2 Table**

Supplementary Figure A. **Discriminant analysis of the bimodal distribution of within-person changes in Abbott SARS-CoV-2 IgG S/C values.**


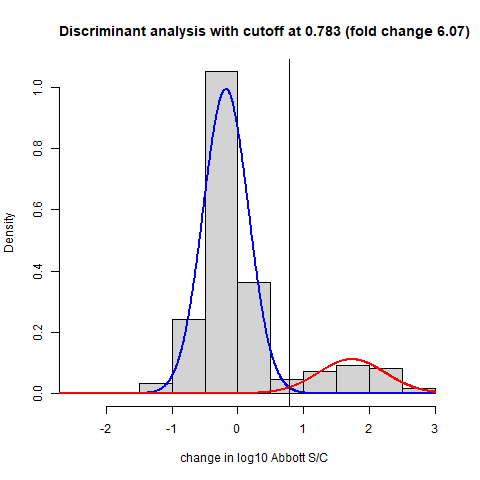


Supplementary Figure B. **Rate of seroconversion, as defined by the fold change shown in Supplementary Figure A, as a function of initial Abbott SARS-CoV-2 IgG S/C values.**


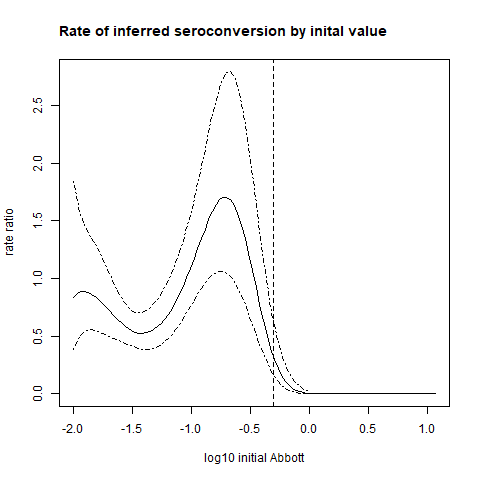


Supplementary Figure C. **SARS-CoV-2** **seroprevalence in 2020-21 implied by the force of infection in Figure B.**


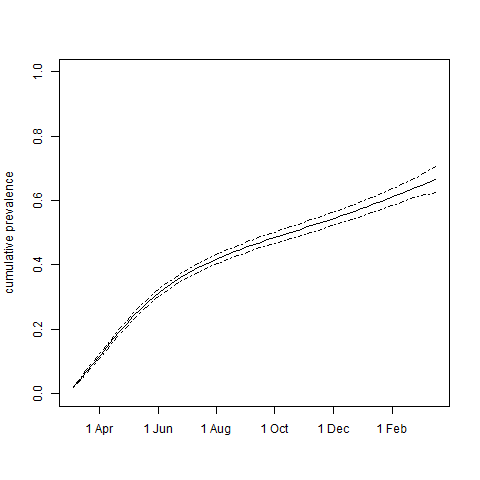


Supplementary Figure D. **Abbott SARS-CoV-2 IgG S/C value plotted against calendar time (2020-21) for first donations within the time period (green) and subsequent donations (brown).** The corresponding lines are the smoothed median SARS-CoV-2 IgG S/C values for the first 95% of first results, and the last 95% of subsequent results. The lines are close together in the overlapping date range, suggesting that the first-time donors did not differ substantially from other donors.


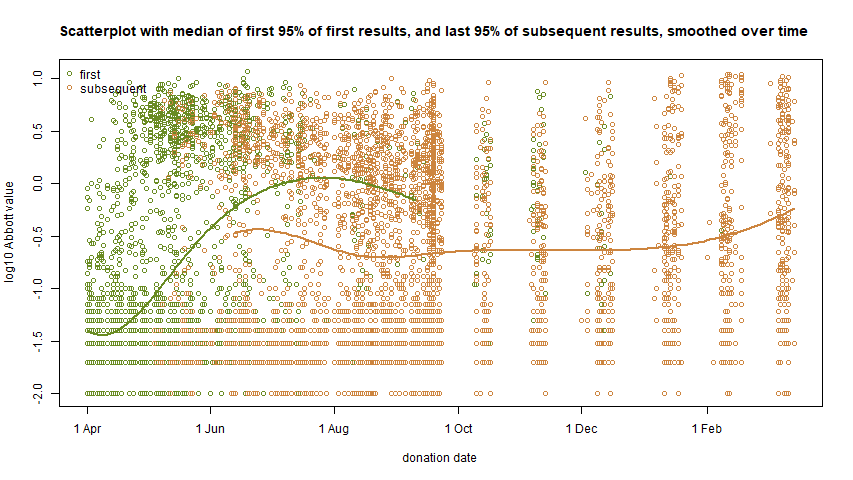


Supplementary Figure E. **Ratio of deaths to cases in the SIVEP-Gripe database.** A rolling weekly average was calculated, according to date of notification for a) cases which were later notified as having a fatal outcome and b) all cases with non-missing outcome. The figure shows the ratio of the former to the latter.


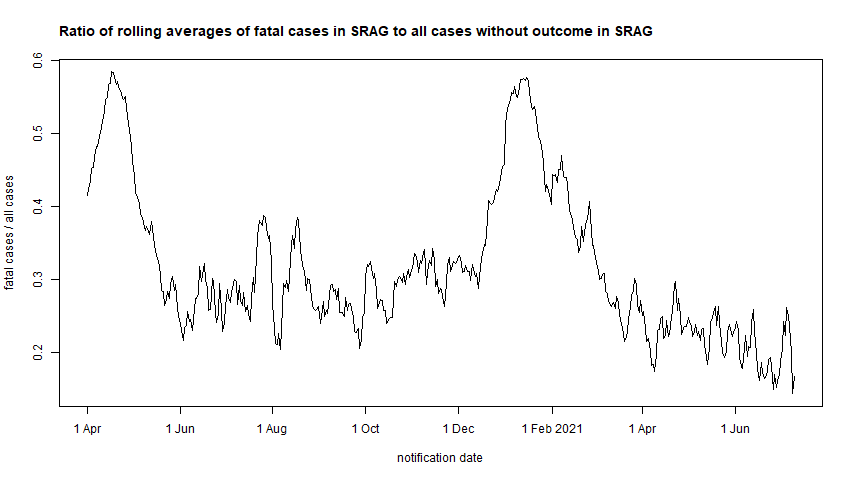

Supplement: S1 File — (DOCX) [file pone.0308319.s002.docx]
